# Modeling the impacts of agricultural best management practices on runoff, sediment, and crop yield in an agriculture-pasture intensive watershed

**DOI:** 10.7717/peerj.7093

**Published:** 2019-07-04

**Authors:** Solmaz Rasoulzadeh Gharibdousti, Gehendra Kharel, Arthur Stoecker

**Affiliations:** 1 Biosystems and Agricultural Engineering, Oklahoma State University, Stillwater, OK, United States of America; 2 Department of Environmental Sciences, Texas Christian University, Forth Worth, TX, United States of America; 3 Department of Natural Resource Ecology and Management, Oklahoma State University, Stillwater, OK, United States of America; 4 Department of Agricultural Economics, Oklahoma State University, Stillwater, OK, United States of America

**Keywords:** Watershed, Sediment, Crop yield, Conservation, SWAT model, Oklahoma, Runoff

## Abstract

Best management practices (BMPs) are commonly used to reduce sediment loadings. In this study, we modeled the Fort Cobb Reservoir watershed located in southwestern Oklahoma, USA using the Soil and Water Assessment Tool (SWAT) and evaluated the impacts of five agricultural BMP scenarios on surface runoff, sediment yield, and crop yield. The hydrological model, with 43 sub-basins and 15,217 hydrological response units, was calibrated (1991–2000) and validated (2001–2010) against the monthly observations of streamflow, sediment grab samples, and crop-yields. The coefficient of determination (*R*^2^), Nash-Sutcliffe efficiency (NS) and percentage bias (PB) were used to determine model performance with satisfactory values of *R*^2^ (0.64 and 0.79) and NS (0.61 and 0.62) in the calibration and validation period respectively for streamflow. We found that contouring practice reduced surface runoff by more than 18% in both conservation tillage and no-till practices for all crops used in this modeling study. In addition, contour farming with either conservation tillage or no-till practice reduced sediment yield by almost half. Compared to the conservation tillage practice, no-till practice decreased sediment yield by 25.3% and 9.0% for cotton and grain sorghum, respectively. Using wheat as cover crop for grain sorghum generated the lowest runoff followed by its rotation with canola and cotton regardless of contouring. Converting all the crops in the watershed into Bermuda grass resulted in significant reduction in sediment yield (72.5–96.3%) and surface runoff (6.8–38.5%). The model can be used to provide useful information for stakeholders to prioritize ecologically sound and feasible BMPs at fields that are capable of reducing sediment yield while increasing crop yield.

## Introduction

Sediments, originating from land use activities including farming and urbanization, constitute one of the major non-point source (NPS) pollutions and have impaired water bodies including wetlands and playas, reduced reservoir capacity and lifespan, threatened drinking water supply, increased water treatment cost, and reduced the overall ecosystem health globally (Abdulkareem et al., 2018; Falconer, Telfer & Ross, 2018; Mateo-Sagasta et al., 2017; Johnson et al., 2012; Hargrove & Devlin, 2010; Simon & Klimetz, 2008; Palmieri, Shah & Dinar, 2001). In the United States of America (USA), more than 50% of water bodies are NPS impaired, with sediment ranking the sixth among the leading causes of water quality impairments (US Environmental Protection Agency, 2016).

The United States Department of Agriculture (USDA) through its conservation programs such as the Great Plains Conservation Program prior to 1996, the Environmental Quality Incentives Program since 1996, the Conservation Stewardship Program, the Conservation Reserve Program, etc. has been providing financial and technical assistance to ranchers and farmers to implement conservation practices and protect water resources while increasing the productivity (Reimer & Prokopy, 2014; Osteen, Gottlieb & Vasavada, 2012; Richardson, Bucks & Sadler, 2008). A modeling study estimated a reduction of sediment load by 3.5% in the US Southern Great Plains region, attributed to the implementation of conservation practices (White et al., 2010). The Great Plains region, characterized by highly intensive agricultural production system in the USA, is subject to water quality issues mostly due to agricultural NPS pollution (Osteen, Gottlieb & Vasavada, 2012). To reduce agricultural NPS pollution, several management practices, including conservation tillage system, are encouraged and adopted in the region. This approach has increased soil organic carbon in the Great Plains (Lewis et al., 2018) and reduced soil erosion significantly (Santhi et al., 2014; Bernard, Steffen & Iiavari, 1996). By replacing only 10–23% of conventional tillage system to conservation tillage system in the Great Plains region, could save one billion tons of soil on highly erodible lands (Bernard, Steffen & Iiavari, 1996). Overall, there has been a substantial decrease in sediment discharge by 145 million metric tons per year in the Missouri-Mississippi River system, the largest river systems in the US, between 1940 and 2007 (Meade & Moody, 2010). Apart from dam construction, the implementation of conservation measures has been attributed for this reduction in sediment discharge (Heimann, Sprague & Blevins, 2011; Meade & Moody, 2010). Despite the annual $5 billion spending to limit agricultural NPS pollution, the water quality issues, particularly agricultural soil loss and deposition, still persist due to agricultural intensification in the USA requiring better land management practices (Heathcote, Filstrup & Downing, 2013).

### BMPs for sediment load reduction

Several studies evaluated the effectiveness of various BMPs in reducing sediment loads from agricultural fields. For example, Zhang & Zhang (2011) reported that the use of sediment ponds as BMPs reduced up to 54–85% sediment from field runoff in Orestimba Creek Watershed, California. Lam, Schmalz & Fohrer (2011) found that the implementation of BMPs related to extensive land use management, grazing management practice, field buffer strip, and nutrient management plan reduced sediment load by 0.8% to 4.9% in a North German lowland catchment. Rousseau et al. (2013) applied vegetated riparian buffer strips, precision slurry application, grassland conversion of cereal and corn fields, and no-till corn in Beaurivage River watershed, Quebec, Canada and found that riparian buffer strips and grassland conversion were highly effective in reducing sediment yield compared to other BMPs. Maharjan et al. (2016) tested three BMPs including split fertilizer application, winter cover crop cultivation, and a combination of the two BMPs in the Haean catchment, South Korea and found that the combination of split fertilizer application and cover crop cultivation resulted the highest positive effect in terms of reduced sediment and nitrate loads and increased crop yield. Teshager et al. (2017) analyzed fourteen scenarios based on systematic combinations of five BMP strategies: fertilizer/manure management, changing row-crop land to perennial grass, vegetative filter strips, cover crops and shallower tile drainage systems, in the Raccoon River watershed in west-central Iowa, USA. Their findings suggest that planting switchgrass in half of the watershed would reduce the sediment load by up to 67% and meet the drinking water standard. Yang et al. (2009) estimated about 51.8–71.4% reduction in sediment loads from the Black Brook Watershed in northwestern New Brunswick, Canada with the implementation of flow diversion terraces.

In this study, we evaluated different agricultural best management practices (BMPs) and estimated changes in sediment load, surface runoff and crop yield in a selected rural agricultural watershed, Fort Cobb Reservoir watershed, located in southwestern Oklahoma, USA. This watershed is reported to have water quality issues related to sediment, despite of BMP implementation in most parts of the watershed for years (Oklahoma Department of Environmental Quality, 2006; Oklahoma Conservation Commission, 2014). Therefore, this study area provides a good site to evaluate how sediment loads alter with the selection and placement of BMPs in the watershed.

**Figure 1:**
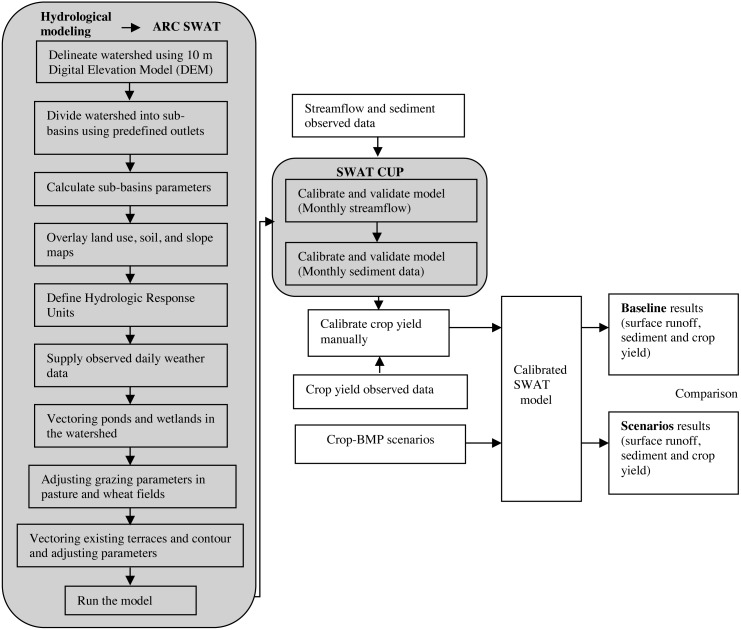
Schematic representation of Best management practices (BMP) implementation in a watershed.

In the Fort Cobb Reservoir watershed, several BMPs such as contour and strip farming, terraces, conversion of crop land to Bermuda pasture, reduced till and no-till farming, drop structures, shelter belts, flood retarding structures, etc. have been currently implemented with about 50% of the cropland under conservation tillage or minimum disturbance tillage (Garbrecht & Starks, 2009). Although hydrological modeling studies of this watershed are available (Storm, Busteed & White, 2006; Moriasi, Starks & Steiner, 2008; Mittelstet, 2015), these studies included very limited BMPs to assess their impacts on water quality. The Oklahoma Department of Environmental Quality recommended a conversion of 50% of the cultivated area in the watershed to no-till practices to control sediment and nutrient loads (Oklahoma Conservation Commission, 2014). Osei et al. (2012) compared the effects of no-till systems on wheat yield with other tillage systems and found that no-till would be more profitable than conventional tillage or the current mix of tillage practices in the watershed. On contrary, the continuous no-till practice showed decreased wheat yield (Decker et al., 2009; Patrignani et al., 2012), which could be due to increased risk of weeds and diseases cycles associated with wheat production (Edwards et al., 2006). To the best of our knowledge, no studies were conducted in the study watershed to estimate changes in sediment loadings due to rotation of no-till winter wheat with other viable crops. Therefore, in this study we estimated the effectiveness of different possible BMPs to reduce sediment loads while increasing the crop yield. To this end, first, a hydrological model of Fort Cobb Reservoir watershed was developed using the Soil and Water Assessment Tool (SWAT) modeling framework (Arnold et al., 1998). The model was calibrated and validated based on streamflow, sediment, and crop yield data. Then, the effectiveness of these BMPs was estimated targeted at sediment reduction and maximization of crop yields. The steps used in this study as illustrated in Fig. 1 are explained in the sections below.

## Materials & Methods

### Study area

**Figure 2:**
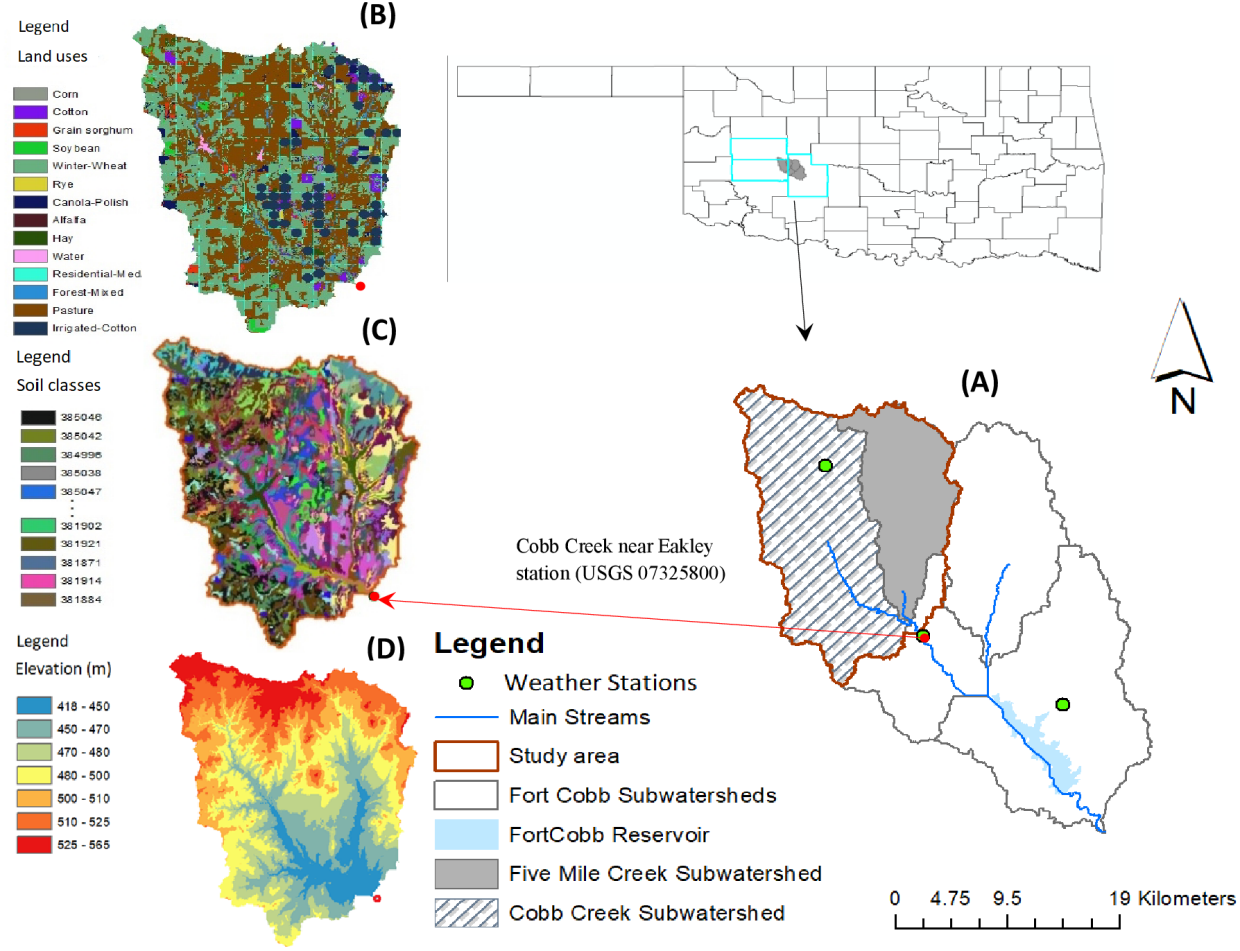
Study area showing (A) Five-Mile Creek sub-watershed (FMC) located within the Fort Cobb Reservoir watershed, (B) Land types, (C) soil classes and (D) elevation maps.

The selected study area is Five-Mile Creek sub-watershed (FMC) located within Fort Cobb Reservoir watershed in southwestern Oklahoma (Fig. 2). FMC has an area of 113.05 km^2^ with land uses comprised of 50% cropland, 41% pastureland and 9% others. The major crops in FMC include 30% winter wheat, 16% cotton (dryland 3.5%, irrigated 12.5%), and grain sorghum (1.5%). Between 1982 and 2016, the study area received 2.2 mm/day precipitation with daily average temperature (15.8 °C), solar radiation (16.9 MJ/m^2^), relative humidity (0.6 fractional), and wind speed (4.3 m/s). The Five-Mile Creek is one of the four tributaries of the Fort Cobb Reservoir (Fig. 2). The reservoir water quality has been of concern for decades and is included in the impaired and threatened waters, 303(d) list, because of high levels of sedimentation, phosphorous, nitrogen, bacteria, and ammonia caused primarily by intensive agriculture and pastoral activities (Oklahoma Conservation Commission, 2009; Oklahoma Department of Environmental Quality, 2014). The 303(d) list comprises those waters that are in the polluted water category, for which beneficial uses like drinking, aquatic habitat, industrial, recreation and use are impaired by pollution. Despite several additional BMPs being implemented, the issues of sedimentation still exist in the study area.

### Hydrological model

The Soil and Water Assessment Tool (SWAT) was employed to construct a hydrological model of the study area using the gaging station (USGS 07325800) of the United States Geological Survey as a watershed outlet. This station is the only available monitoring station with continuous records of streamflow. It receives runoff from two sub-watersheds- Cobb Creek and FMC sub-watersheds (Fig. 2). A ten-meter Digital Elevation Model was used for watershed delineation, stream network creation and topographic information. The study area was divided into spatially related 43 sub-basins with an average area of 8 km^2^ (0.2–28 km^2^). The watershed topography was grouped into four slope classes of 0–2%, 2–4%, 4–6%, and >6%. Existing waterbodies including ponds in the watershed were obtained from US Fish and Wildlife Service (2014) and modeled these waterbodies as ponds in each sub-basin (Appendix A). The SSURGO soil database (Soil Survey Staff, 2015), the finest resolution soil data available, was used to define soil attributes in the watershed (Appendix B). The land use data were obtained from the 2014 crop data layer (USDA-NASS, 2014). The cultivated land cover types were further separated into irrigated and non-irrigated lands based on the locations of the center pivot irrigation circles. These locations were identified from the 2014 one-meter resolution aerial images (https://datagateway.nrcs.usda.gov/). We found 30 pivot circles encompassing 13.7 km^2^ (12.1%) of irrigated land dedicated for cotton production in the FMC sub-watershed. An Overlay of land use, soil and slope with respective SWAT threshold percentages of 10% for land, 10% for soil and 20% for slope in each sub-basin resulted into 15,217 Hydrologic Response Units (HRUs). An HRU in SWAT captures watershed diversity by combining similar land, soil and slope areas in each sub-basin. In SWAT, loadings of water, sediments, and crop yield are calculated first at HRU level, summed at each sub-basin and then routed to the watershed outlet.

These HRUs were assigned agricultural BMPs (conservation tillage, no-till, contouring, crop rotation, and conversion to pasture—Bermuda grass) that are most commonly practiced in the study area. Existing contour in the study watershed were identified by using aerial photographs (Barber & Shortridge, 2005). The broken terraces were recognized using two-meter LiDAR drainage lines from satellite imagery (https://gdg.sc.egov.usda.gov/Catalog/ProductDescription/LIDAR.html). The HRUs with more than 65% contour were classified as being terraced with contour farming. It was found that 8 km^2^ of FMC were terraces and contour without breaking, which modeling existing terraces and contours resulted into 28% reduction in sediment.

Information about tillage type and fertilizer application for the selected crops was obtained from relevant literature (Storm, Busteed & White, 2006; Oklahoma Department of Environmental Quality, 2006) and consultation with local Oklahoma State University Cooperative Extension Service and Conservation District personnel (Appendix C.1–9). Additionally, cattle information including cattle stocking rate (0.5 head/ha), consumed biomass (3 kg/ha/day), trampled biomass (0.47 kg/ha/day) and deposited manure (1.5 kg/ha/day) were obtained from other sources (USDA-NASS, 2012; Storm, Busteed & White, 2006) and used in the model.

The current climate pattern (1982–2016) in the watershed was represented by six climate variables: precipitation, minimum temperature, maximum temperature, solar radiation, relative humidity and wind speed. The climate data at daily scale were collected from a combination sources including the USDA Agricultural Research Service (USDA-ARS) (http://globalweather.tamu.edu/), the Oklahoma MESONET (https://www.mesonet.org/).

### Model calibration and validation

First, the model was calibrated manually to improve the model performance based on operation management parameters and associated cropping schedules and then automated iterative calibration was performed using SWAT-CUP tool (Abbaspour et al., 2007a; Abbaspour et al., 2007b) for three important components: streamflow, sediment, and crop yield. Crop operation management parameters and associated cropping schedules were adjusted manually. Model sensitivity was tested prior to model calibration to determine the most sensitive parameters. Observed data on streamflow, crop yields and sediment loads from 1990 to 2010 were used for model calibration and validation. Three different statistical matrices—coefficient of determination (*R*^2^), Nash-Sutcliffe efficiency (NSE) and percent bias (PB) were used to evaluate the model performance.

### Streamflow

Monthly streamflow observed at the USGS gaging station—Cobb Creek near Eakely gage (USGS 07325800) for a ten-year period (1991–2000) was used for model calibration. Prior to model calibration, the sensitivity of the model to streamflow was tested in SWAT-CUP for 17 parameters. The *p*-value and t-state indicators were used to identify the most sensitive parameters in the watershed. The smaller the *p*-value and the larger the absolute value of t-state, the more sensitive the parameter is. The six parameters related to water balance: Curve number (CN), soil evaporation compensation factor (ESCO), groundwater delay (GW_DELAY), deep aquifer percolation fraction (RCHRG_DP), Manning’s n value for the main channel (CH_N2), and available water capacity of soil layer (SOL_AWC) were found to be the most sensitive (Appendix D), similar to what other studies found (Moriasi, Starks & Steiner, 2008; Storm, Busteed & White, 2006).

**Figure 3:**
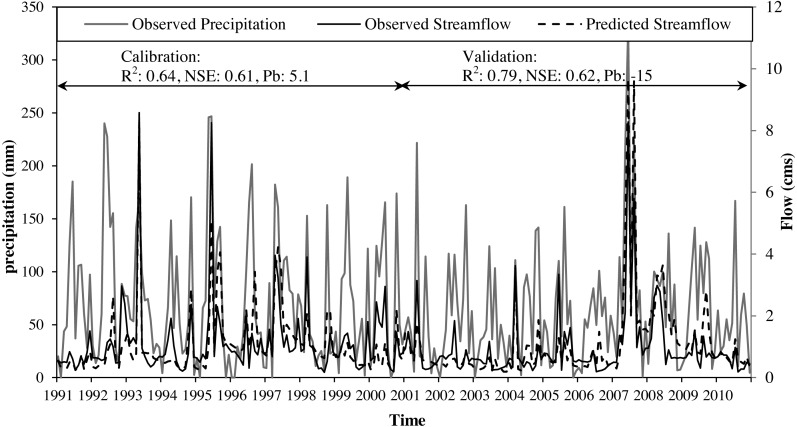
Calibration and validation monthly time series (2000–2010) for average monthly precipitation along with observed and SWAT predicted streamflow at the Cobb Creek near Eakley, Oklahoma gauging station.

According to Moriasi et al. (2015), model performance can be judged “satisfactory” for flow simulations if daily, monthly, or annual *R*^2^ > 0.60, NSE >0.50, and PB ≤±15% for watershed-scale models. The model was calibrated satisfactorily for streamflow with values of *R*^2^ (0.64) and NSE (0.61) and PB (5.1%) (Fig. 3). The validation of the model with an independent set of monthly observed streamflow at the same gage station for a different ten-year period (2001–2010) indicated a robust model performance with values of *R*^2^ (0.79) and NSE (0.62) and PB (−15%) (Fig. 3). Calibrated parameters and their final value ranges are listed in Table 1.

**Table 1:** Streamflow and sediment calibration parameter values in study area.

**Component**	**Parameter**	**Parameter value range**	**Calibrated value**
Streamflow	V__GWQMN.gw	0.20_0.60	0.60
	V__GW_REVAP.gw	0.02_0.03	0.02
	V__REVAPMN.gw	0.50_1.50	1.38
	V__RCHRG_DP.gw	0.10_0.50	0.47
	V__GW_DELAY.gw	320_390	376
	R__CN2.mgt	−0.16_−0.13	−0.13
	V__ALPHA_BF.gw	0.80_1.00	0.95
	V__ESCO.hru	0.80_0.90	0.83
	V__EPCO.bsn	0.10_0.60	0.30
	V__CH_K1.sub	0.00_0.40	0.09
	V__SURLAG.bsn	0.50_4.00	3.05
	V__EVRCH.bsn	0.00_0.50	0.34
	V__TRNSRCH.bsn	0.00_0.10	0.10
	V__ALPHA_BNK.rte	0.60_1.00	0.84
	R__SOL_AWCsol	−0.02_0.06	0.04
	V__CH_N2.rte	0.05_0.30	0.18
	V__CH_K2.rte	1.85_2.15	1.98
Sediment	R__USLE_P.mgt	−1.000_0.000	−0.240
	R__SLSUBBSN.hru	0.000_0.230	0.217
	R__USLE_Ksol	−0.500_0.300	−0.247
	V__RSDCO.bsn	0.010_0.100	0.083
	V__BIOMIX.mgt	0.000_0.300	0.297
	V__SPCON.bsn	0.000_1.000	0.009
	V__SPEXP.bsn	1.000_2.000	1.714
	V__CH_ERODMOrte	0.050_0.700	0.355
	V__CH_COV1.rte	0.001_0.800	0.518
	V__CH_COV2.rte	0.001_0.800	0.332

**Notes.**

“R” before the parameter name stands for relative change (the parameter is multiplied by 1 + value); “V” stands for replacement (the parameter is replaced by a value within the range).

### Sediment

**Figure 4:**
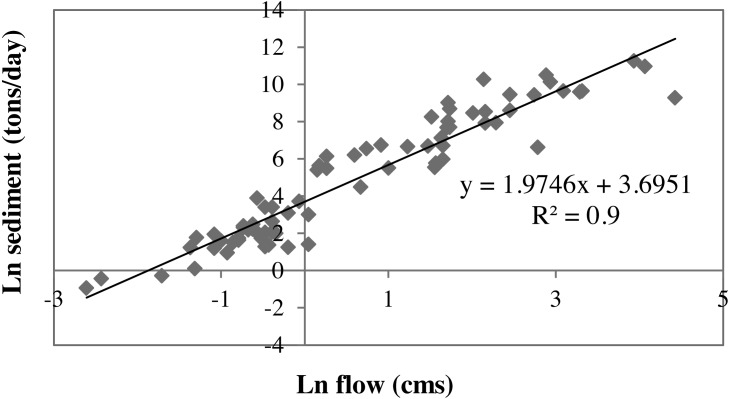
Observed daily discharge and observed daily suspended sediment concentration trend.

**Figure 5:**
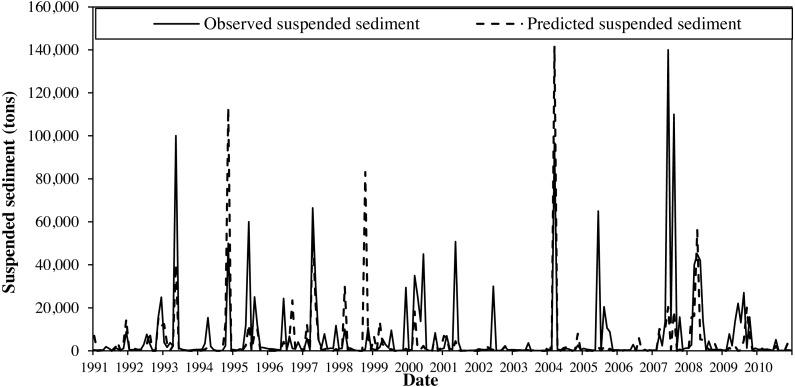
Calibration and validation monthly time series (2000–2010) for observed and SWAT simulated suspended sediment concentration at the Cobb Creek near Eakley, Oklahoma gauging station.

Suspended sediment was calibrated for ten years (1991–2000) and validated for another ten years (2001–2010) at the watershed outlet. For this, grab suspended sediment sample data that were available from 2004 to 2012 (usually 1 to 3 samples per month with a few months missing) was used. This grab sample data provided us an opportunity to estimate sediment loads for the time period that lacked observations using sediment rating curve method as suggested by Horowitz (2003). This method is a regression relationship between the observed streamflow and sediment data used popularly to generate sediment information for missing period in many modeling studies (Salimi et al., 2013; Shabani & Shabani, 2012; Jothiprakash & Garg, 2009; Sarkar et al., 2008; Gray & Simões, 2008). A strong correlation (*R*^2^ = 0.9) between the observed grab sample sediment data and runoff in the study watershed (Fig. 4) was observed. We used this regression relationship to estimate the missing sediment data for this study. Then these data were used to calibrate the model by modifying ten parameters that were related to sediment load (Storm, Busteed & White, 2006; Moriasi, Starks & Steiner, 2008). The model calibration with values of *R*^2^ (0.35), NSE (0.30) and PB (<20%), (Fig. 5) and validation with values of *R*^2^ (0.43), NSE (0.33) and PB (<55%) (Fig. 5) was considered acceptable for this study. Calibrated parameters and their final value ranges are listed in Table 1.

We found that the largest errors in sediment prediction were associated with errors of peak flow estimation. This could be due to the “second storm effect” problem in hydrological models, including SWAT (Abbaspour et al., 2007a; Abbaspour et al., 2007b). The first storm event causes a larger sediment transport and makes remaining surface layers difficult to mobilize. As a result, the second and third storm events regardless of their event sizes, will result in smaller sediment loads. For this study area, the “second storm effect” was not tested since there were no observed sediment data representing flood events (May 1993, June 1995, June 2007) during model calibration-validation period. The simulated sediment data failed to accurately capture these events, resulting uncertainty in sediment calibration. The over-and under-estimation of sediment during flood events was reported in other SWAT based studies (Oeurng, Sauvage & Sánchez-Pérez, 2011).

### Crop yield

**Figure 6:**
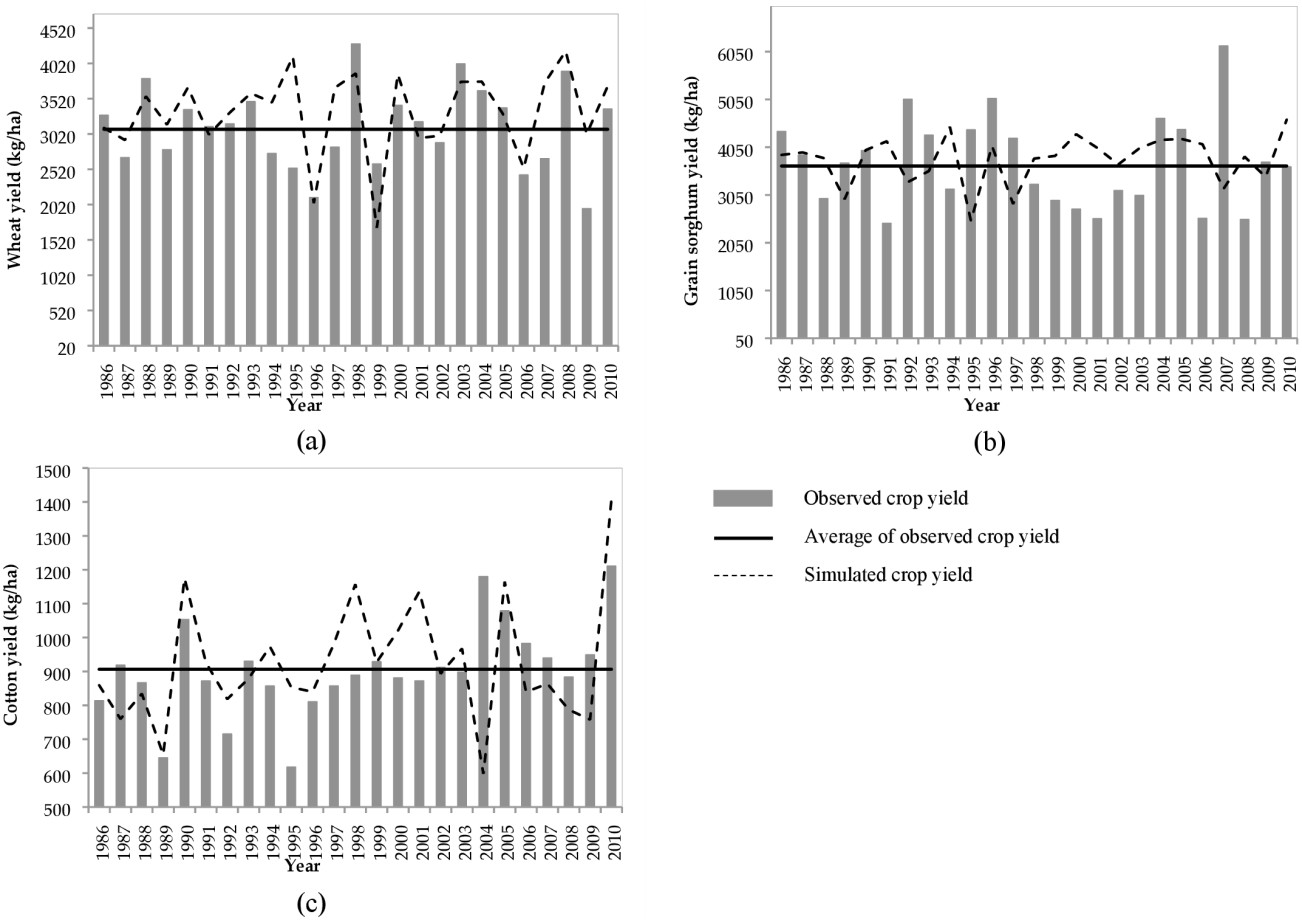
Observed and simulated average annual yields of (A) winter wheat, (B) grain sorghum, and (C) cotton in the study area.

Crop yield and biomass production affect watershed hydrology through altered erosion and water balance (Hu et al., 2007; Ng et al., 2010; Andersson et al., 2011; Nair et al., 2011). A combination of the Oklahoma State University variety trial data from 2001 to 2016 (http://croptrials.okstate.edu/), and the county level data (1986–2005) obtained from the USDA National Agricultural Statistics Service were used to calibrate yield of crops (winter wheat, grain sorghum, cotton- both dry and irrigated) (USDA-NASS, 1986 to 2005, http://digitalprairie.ok.gov/cdm/ref/collection/stgovpub/id/11177). The variety trial crop yields were collected from sites in seven Oklahoma counties (Apache, El Reno, Homestead, Chickasha, Altus, Tipton, and Thomas) that are located within and nearby the study area. A list of crop yield parameters with their initial and calibrated values is provided in Appendix E.1 and E.2. In this study the PB was used as an indicator to compare the SWAT simulated yield with the observation. Ten crop model parameters were selected (Appendix E.1 and E.2) and their associated value ranges were set based on recommendation made by Sinnathamby, Douglas-Mankin & Craige (2017) and Nair et al. (2011). The values were then manually adjusted until the percentage bias (PB) for the modeled crops reached satisfactory values: cotton (−4.5%), grain sorghum (−27.3%) and winter wheat (−6.0%) during the 1986–2010 model simulation period (Fig. 6).

### Agricultural best management practices scenarios

Studies identified sedimentation as one of the water quality issues in the region with the associated ecological and economic impacts (Zhang et al., 2015). As a result, various agricultural BMPs have been implemented in the watershed to abate sediment loading and transport (Becker, 2011). Despite these efforts, there are still soil erosion problem in agricultural fields causing degraded water quality (Oklahoma Department of Environmental Quality, 2014).

Often, conservation tillage and no-till practices can be employed to improve the success of new cropping systems and help assure the sustainability of the land. No-till cropping systems in Oklahoma have proved important resources for the economic viability of producers and landowners operations (Malone et al., 2007). Conversion to no-till practices on at least half of the cultivated area in the study watershed was one of the recommendations to reduce sediment and nutrient loadings for this Watershed (Oklahoma Conservation Commission, 2014). Conservation practices such as contour and strip farming, terraces, conversion of crop land to Bermuda pasture, reduced till and no-till farming, drop structures, shelter belts, flood retarding structures, etc. have been currently implemented in the study region as the effective BMPs for mitigating NPS pollution (Garbrecht & Starks, 2009). However, records detailing types and time of installation of these management practices prior to the 1990s are not readily available in either the state offices of the Natural Resources Conservation Service (NRCS) or the local conservation districts. According to Garbrecht & Starks (2009), 80%–90% of cropland in the study area that needed terraces, has been terraced over the last 50 years. Over the last decade, about 50% of the cropland was in conservation tillage or minimum disturbance tillage. In addition to these management practices, gully reshaping and grad stabilization structures were implemented by conservation funds. Other conservation practices have been implemented by farmers without cost sharing assistance. Also, some selected channel bank sections were stabilized and some channels have been fenced to prohibit cattle from eroding banks, small impoundments were constructed, and a number of gravel roads were paved to control cropland erosion in this watershed. Despite these efforts, there are issues of NPS pollution in the region (Oklahoma Department of Environmental Quality, 2006; Oklahoma Conservation Commission, 2014). Therefore, we developed five scenarios that reflect the commonly used agricultural BMPs in the study area and throughout the Great Plains region (Table 2). These BMPs included practices of conservation tillage and no till on both contouring and no-contouring along with the rotation of winter wheat with other crops. The BMPs were applied to three major crops- cotton, grain sorghum and winter wheat. Because of weed and disease problems associated with continuous no-till wheat, wheat was rotated/cover cropped with canola, cotton and grain sorghum. A combination of land use and these five scenarios resulted into 22 SWAT model simulations. In scenarios 1–4, the study area was simulated for one crop at a time by converting all crops into one (for example, all crops converted to wheat and so on). In scenario 5, all the cropland in the study area was converted to Bermuda grass because of its popularity in the study watershed (Moriasi, Starks & Steiner, 2008).

**Table 2:** Agricultural Best Management Practices (BMPs) scenarios simulated for, cotton, grain sorghum and winter wheat to evaluate their impacts on hydrology, sediment and crop yield in the study area.

**Code**	**BMP Scenario**	**Description**
BL	Baseline	Simulation under the calibrated and validated model with 14 land uses, 8 km2 FMC under contour farming
S1	Conservation tillage and strip cropping	BMP applied to cotton, grain sorghum, and winter wheat. No changes made to hay and alfalfa. Data obtained from NASS (2014), Storm et al. (2003) and Storm, Busteed & White (2006). Total three simulations, one for each crop.
S2	Conservation tillage on contour	Applied contour on scenarios 1; 97 km2 additional contour as compared to the baseline scenario. Resulted three simulations, one for each crop.
S3	No-till and strip cropping	All tillage practices were removed while management practices were kept the same; applied to cotton, grain sorghum and winter wheat. Because of weed and disease problems associated with continuous no-till wheat, wheat was rotated/cover cropped with (i) canola, (ii) cotton and (iii) grain sorghum. Total five simulations, one for each crop.
	No-till wheat in rotation with canola	
	No-till wheat as a cover crop for cotton	
	No-till wheat as a cover crop for grain sorghum	
S4	No-till on contour	Applied contour on Scenario 3. Resulted five simulations, one for each crop.
S5	Conversion to pasture	All crops were converted to Bermuda grass pasture. A combination of three grazing start months (May, June and July) and two stocking rates (1,200 and 1,600 kg) were applied. Total of six simulations.

**Notes.**

Details of each scenario are provided in Appendix F.

## Results

### Surface runoff

**Figure 7:**
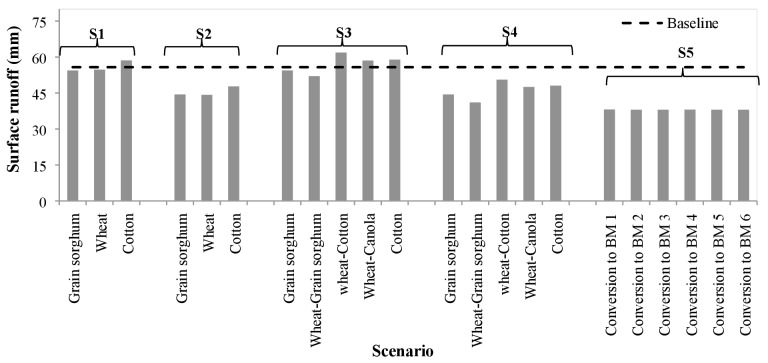
Changes in surface runoff generation under different scenarios of Best Management Practices.

All five scenarios except for S3 with wheat-cotton and wheat-canola rotations and cotton in S1 and S3 decreased surface runoff compared with the baseline scenario (Fig. 7). When contouring was applied in conservation tillage (S2), surface runoff reduced by 18.4% for cotton and grain sorghum and by 19.2% for winter wheat. Similarly, implementation of contouring on the existing no-till BMP (S4) led to surface runoff reduction by 18.4% (cotton and grain sorghum) and 19.4% for wheat compared to the no-till BMP (S3). Between the three major crops in scenarios 1 to 4, grain sorghum was the least runoff generator followed by winter wheat and cotton. When all crops were converted to Bermuda grass (S5) surface runoff reduced by 31.7% as compared to rest of the scenarios. Application of different grazing operations and stocking rates in S5 resulted virtually the similar runoff generation (37.96–38.08 mm) with less than one-third of a percentage point difference between them. Of the 22 combinations of agricultural BMPs simulated in all five scenarios, wheat rotated with cotton under no-till resulted the highest runoff followed by wheat rotated with canola. We found that there was virtually no change in surface runoff between the conservation and no-till systems. However, the implementation of contouring reduced surface runoff in both conservation and no-till systems.

### Sediment

**Figure 8:**
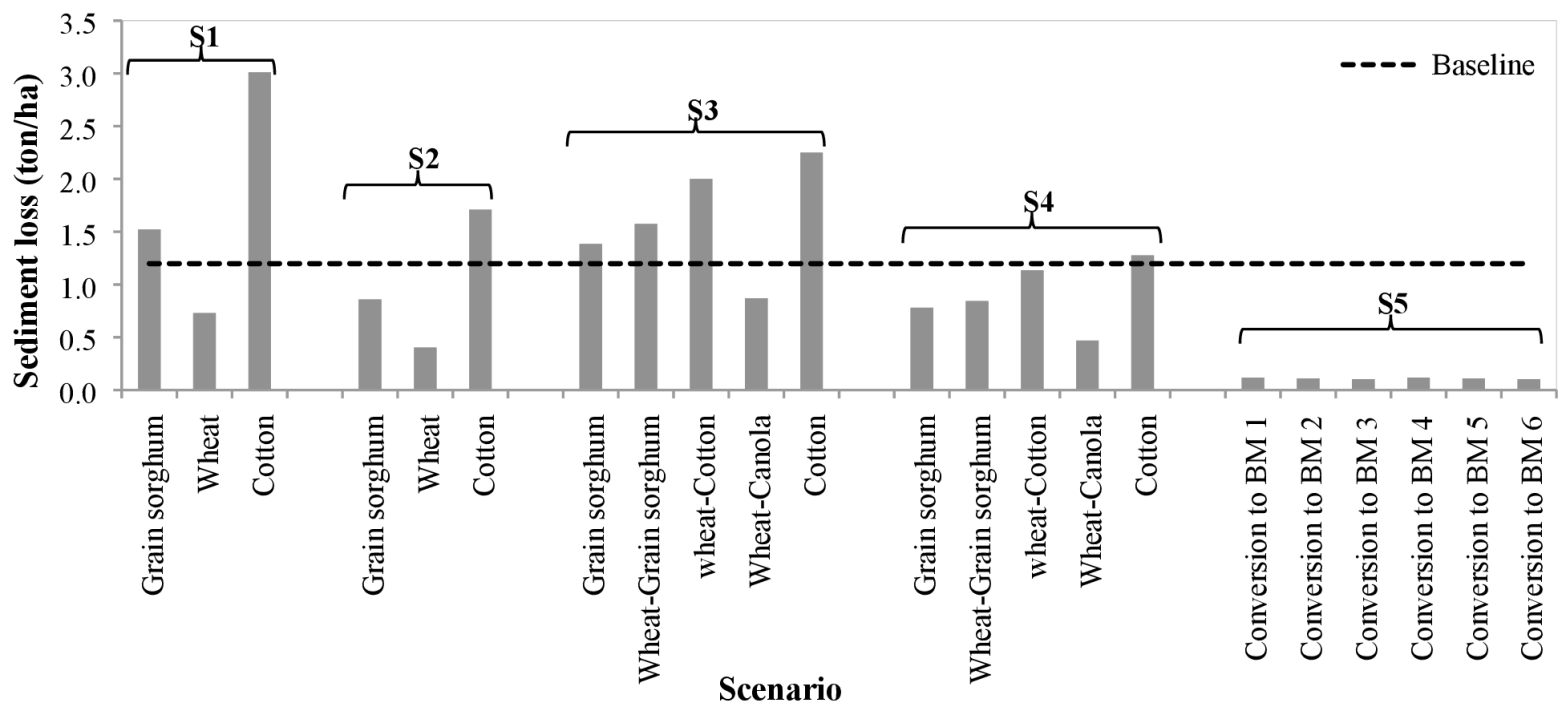
Average annual sediment loss (tons per hectare) under each five agricultural Best Management Practices scenarios compared with the baseline scenario.

We found that implementation of contouring on conservation tillage (S2) and on no-till (S4) reduced sediment loss nearly by half (Fig. 8 and Table 3). Between all 22 combinations of BMPs, cotton was the lead contributor to sediment. For cotton, contouring on no-till practice generated the least sediment (1.27 tons/ha) while the conservation tillage with no contouring released most sediment (3.01 ton/ha). Wheat’s contribution to sediment loss was as half as that of grain sorghum and one-fourth of that of cotton (S1–S4). Wheat, under the conservation tillage with contour (S2), was the least contributor of sediment (0.4 ton/ha). Rotating wheat with canola was found to be the most effective in controlling sediment loss under no-till system with only 0.87 ton/ha loss as compared to wheat as a cover crop for cotton (2.0 ton/ha) and grain sorghum (1.57 ton/ha). Converting all crops to Bermuda grass pasture with combinations of different grazing time and stocking rate (S5) released only 0.10 to 0.12 ton/ha sediment. We found virtually no difference in simulated sediment loss between the combination of grazing timings and stocking rates applied.

**Table 3:** Sediment reduction in percentage as a result of contouring on conservation tillage and no-till practices for cotton, grain sorghum and winter wheat.

**Grain sorghum**		**Cotton**		**Wheat**			
Conservation tillage	No-till	Conservation tillage	No-till	Conservation tillage	No-till (In cover cropping/rotation with)		
					Grain sorghum	Cotton	Canola
44	44	45	46	43	46	43	43

**Figure 9:**
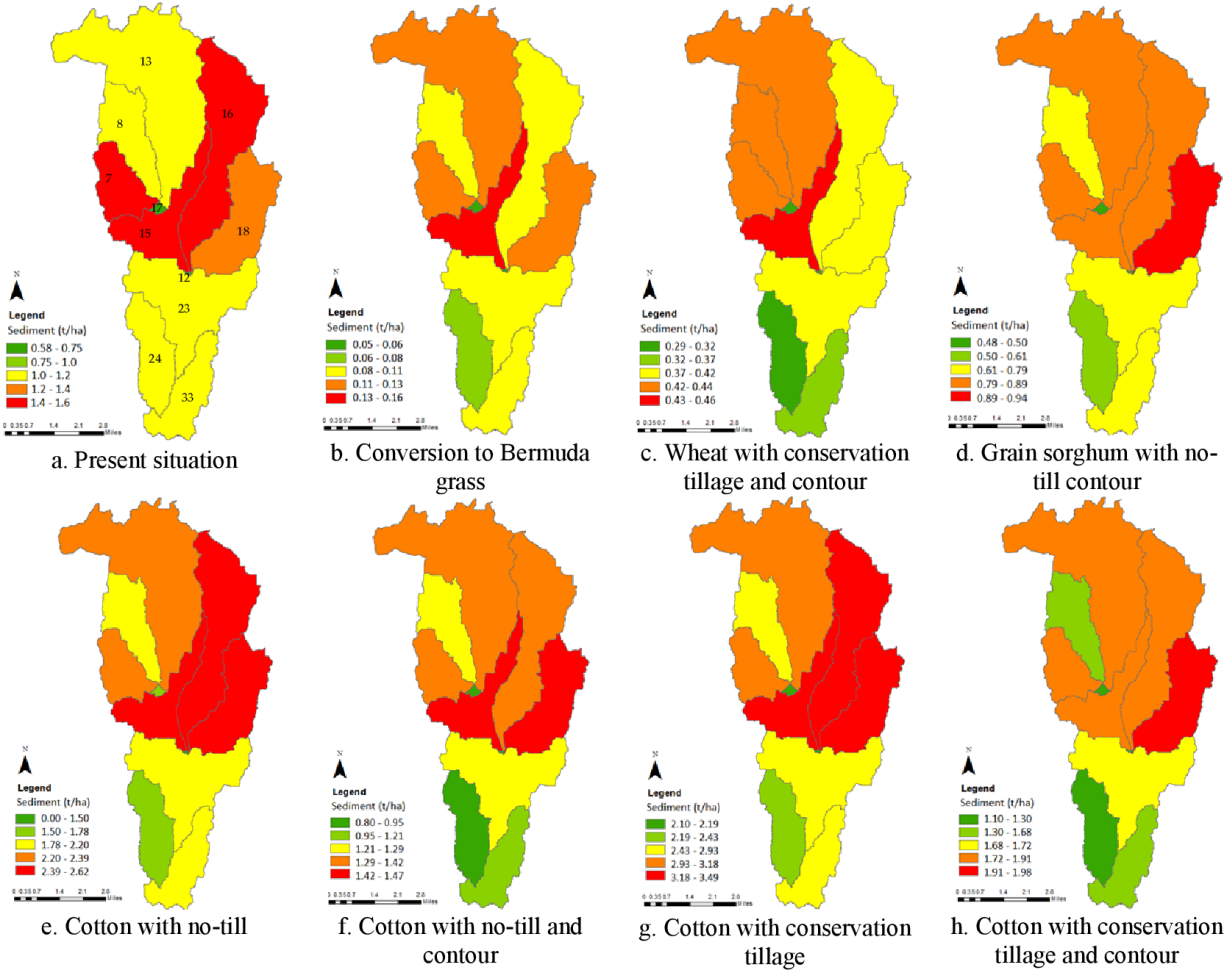
Sub-basin level SWAT simulated sediment loadings (tons/ha) in Five-Mile Creek sub-watershed under different BMP scenarios. (A) Present situation. (B) Conversion to Bermuda grass. (C) Wheat with conservation tillage and contour. (D) Grain sorghum with no-till contour. (E) Cotton with no-till. (F) Cotton with no-till and contour. (G) Cotton with conservation tillage. (H) Cotton with conservation tillage and contour.

In the business-as-usual baseline scenario (BL), the four out of 11 sub-basins (#7, 15, 16, 18) generated sediments at an average of 1.2–1.5 ton/ha (Fig. 9A). These four sub-basins have erosive soil texture (fine sandy loam and silty clay loam) with wheat (28.5%) and cotton (18.5%) as major crops. The amount and location of sediment loadings varied between the scenarios. For example, 90% of sediment load was reduced when the crops were converted to Bermuda grass (Fig. 8B), while the sediment load was increased by 76% and 135% with cotton under no-till and under conservation tillage respectively (Figs. 9E–9H).

### Crop yield

**Figure 10:**
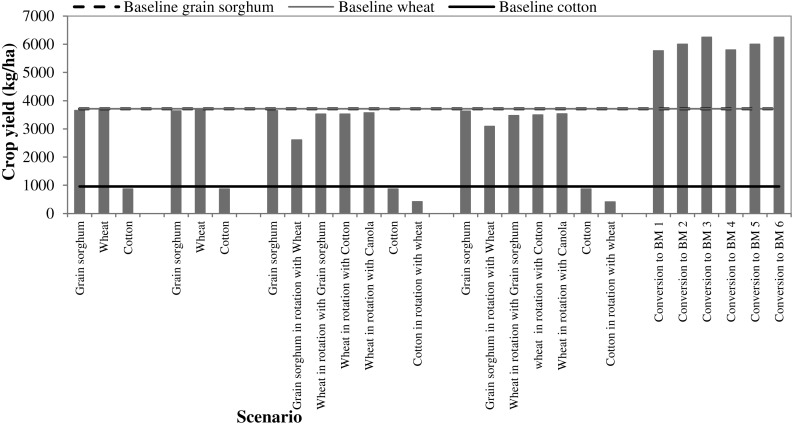
Crop yields under different scenarios of Best Management Practices.

We found no significant effect of contouring and tillage systems on the simulated yields of cotton, grain sorghum and winter wheat. However, we found differences in yields of these crops when they were used as cover crop or in rotation. For example, under the no-till practice, the yield of grain sorghum when wheat was used as a cover crop decreased by 28.4% (S3) and once there was no-till plus contour farming it decreased by 14.8% (S4). It was found that covering/rotation with winter wheat resulted into reduced yield for both cotton and grain sorghum regardless of contouring (S3 and S4). When covering/rotated with winter wheat, cotton yield decreased by 52% with or without contouring while grain sorghum yield decreased by 28.4% (no contour) and by 14.8% with contour (S3 and S4). This decreased yield is attributed to the presence of wheat residues and lack of available soil moisture for the second crop. We found that cotton yield decreased more than that of grain sorghum when wheat was used as a cover crop. We found virtually no effect of stocking rate and grazing start months on pasture yield (Fig. 10).

## Discussion

Five Mile Creek is one of the main contributing sub-watersheds of the Fort Cobb Reservoir watershed. It is a typical example of agriculture-pasture intensive watershed in the US Great Plains that may present a test bed for simulating the impacts of agricultural activities in combination with various BMPs on crop yield, water quality and quantity. In order to reduce erosion in the Fort Cobb Reservoir watershed, several BMPs and conservation measures including terraces, changing cropping patterns, and progressive adoption of no-till and conservation tillage systems among others have been implemented (Oklahoma Conservation Commission, 2014). There are conservation programs with financial and technical assistance available to install new tillage or cropping systems in the study region (USDA, Farm Service Agency, 2016). Some farmers have converted the highly erosive parts of their crop land to Bermuda grass pastureland (USDA-FSA, 2015). These initiatives reduced sediment loadings by three to five times as compared to the time prior to 1963 (Zhang et al., 2015). Garbrecht, Starks & Moriasi (2008) stated that there was substantial reduction in sediment yield in the Five Mile Creek sub-watershed in the second half of the 20th century mainly due to conversion of cropland to pasture land. However, the sediment loads in the study area are still high and need to be reduced (Oklahoma Department of Environmental Quality, 2014). Therefore, in this study, we evaluated the effectiveness of agricultural BMPs on surface runoff, and crop and sediment yields.

### Impacts of contouring and tillage on runoff, sediment and crop yield

Contouring and terracing are popularly used practices to control erosion in the study region (Garbrecht & Starks, 2009). We found that contouring with either conservation tillage or no-till farming prevented sediment yield by almost half while the surface runoff was reduced by at least 18% in the watershed. Compared to the conservation tillage practice, no-till farming decreased sediment yield by 25.3% and 9.0% for cotton and grain sorghum respectively. In several other watersheds, no-till practice was found to generate less sediment yield (Dickey et al., 1983; Olson, Ebelhar & Lang, 2010; Parajuli et al., 2013; Sharpley & Smith, 1994). We found virtually no difference in surface runoff and yields of cotton and grain sorghum between the conservation till and no till practices similar to what was observed by Sharpley & Smith (1994) in the Southern Plains region of Kansas, Oklahoma, and Texas. However, Fawcett, Christensen & Tierney (1994) in their review of several paired watersheds reported that conservation tillage usually led to reduced sediment and surface runoff.

### Impacts of crop rotation/cover on runoff, sediment and crop yield

We found differences in runoff and crop yields as a result of crop rotation. Surface runoff decreased for sorghum (−4.6% vs. −8.1% with contour) and increased for cotton (+5% regardless of contouring) when these crops were rotated with winter wheat. The effect of wheat as cover crop for grain sorghum generated lowest runoff followed by its rotation with canola and cotton regardless of contouring. Sediment yield increased for sorghum (13.7% vs. 8.0% with contour) and it decreased for cotton (11.0% regardless of contouring) when these crops were rotated with winter wheat. The sediment yield was the highest for cotton followed by grain sorghum and canola when rotated with winter wheat regardless of contouring.

Yields of both cotton and grain sorghum decreased when winter wheat was used as a cover crop. Cotton yields decreased by 52.2% regardless of contouring (51% dry land cotton and 62% irrigated lands cotton). Grain sorghum yields decreased by 28.4% vs. 14.8% under contour farming. Winter wheat yield remained virtually the same when rotated with canola and used as a cover crop for grain sorghum and cotton regardless of contouring. Osei (2016) applied three conservation practices in the Fort Cobb River watershed to find the optimal distribution of conservation practices and indicated that no-till winter wheat production in central Oklahoma results in a small cost reduction while maintaining yields and is the win-win option. But since continuous no-till wheat is not possible because of weeds and other disease, it is not the good scenarios for adoption.

### Impacts of crop conversion to pasture on runoff and sediment yield

We found that converting all the crops in the watershed into Bermuda grass would significantly reduce runoff by 6.8 to 38.5%, and decrease sediment loss by 72.5 to 96.3%. We did not find major difference on surface runoff and sediment loss due to two different stocking rates (1,200 and 1,600) on three grazing timings. Although conversion to pasture may be costly without government incentives, it leads substantial and consistent reductions in all environmental indicators through reduced sediment and nutrient losses (Osei, 2016).

Success of the BMP installation in the FCR watershed is of interest to many groups because erosion and transport of sediment and associated nutrients are common problems in the surrounding agricultural watersheds (Becker, 2011). Moreover, state and federal funding has supported the implementation of conservation practices in the watershed (Steiner et al., 2008). Boyer, Tong & Sanders (2017) stated that farming experience, gender and attitudes towards soil and water conservation increases the total number of practices adopted. According to Tong, Boyer & Sanders (2017), negative externalities are the main challenges for adoption of conservation practices in the FCR watershed, and this point indicates the need for new extension educational efforts, economic incentives from government, and research efforts to reduce to negative externalities. These negative effects of sediment and other NPS pollutions are not paid for by the producers and landowners. Instead, downstream users (e.g., recreationists and municipal systems) face the costs. The principal approach for adoption of conservation practices for reduction of NPS pollution from agricultural fields in the USA is subsidizing adoption of conservation practices instead of taxing inputs like sediment and phosphorous. So, there should be motivations from government for landowners and producers to implement conservation practices. In this regard, apart from the environmental impact of different agricultural BMPs, there should be economic consideration of these management practices for selecting the most cost efficient BMPs since funding agencies are better appreciating the link between farm economics and producer adoption of the conservation practices.

## Conclusions

We employed SWAT model to estimate changes in surface runoff, sediment load and crop-yield under five different scenarios of agricultural BMPs in an agriculture-pasture intensive watershed located in southwestern Oklahoma. We found that no-till system released less sediment load than conservation tillage system. Compared to the conservation tillage practice, no-till system decreased sediment load by 25.3% and 9.0% for cotton and grain sorghum respectively. The contour farming with either conservation tillage or no-till practice significantly reduced sediment load. Similarly, contour tillage practices reduced surface runoff by more than 18% in both conservation tillage and no-till practices for all crops. We found varying impacts of wheat used as a cover crop on surface runoff, sediment load and crop yield. We found decreased runoff for grain sorghum and increased runoff for cotton when wheat was used as a cover crop with no-till system. However, we found increase in sediment load for both cotton and grain sorghum when no-till wheat was used as a cover crop. A hypothetical conservation scenario that converted all crops to Bermuda grass pasture land reduced runoff sediment yield significantly but the practicality of this scenario can be realized only with financial incentive programs.

##  Supplemental Information

10.7717/peerj.7093/supp-1 Appendix S1 Reservoir and Ponds Information in the SWAT model

10.7717/peerj.7093/supp-2 Appendix S2 Soil characteristics for each soil ID (SSURGO database)

10.7717/peerj.7093/supp-3 Appendix S3 Conventional (reduced) tillage for different crops and pasture

10.7717/peerj.7093/supp-4 Appendix S4 Global sensitivity analysis results of SWAT-CUP for streamflow

10.7717/peerj.7093/supp-5 Appendix S5 Crop yield calibration parameters

10.7717/peerj.7093/supp-6 Appendix S6 Definition of modeling of crop rotation in SWAT

## Additional Information and Declarations

### Data Availability

The following information was supplied regarding data availability: The raw measurements are availables in the Appendices (Supplemental Files). The raw data in Appendix A shows all reservoir and ponds Information in the SWAT model. Appendix B presents soil characteristics for each soil ID (SSURGO database). Conventional (reduced) tillage for dryland and irrigated crops and pasture and rotation of crop are available in Appendix C. Global sensitivity analysis results of SWAT-CUP for streamflow are available in Appendix D. Appendix E shows the crop yield calibration.
