# Supplementary material for: Modeling the impacts of agricultural best management practices on runoff, sediment, and crop yield in an agriculture-pasture intensive watershed
Source: PeerJ. 2019 Jul 4;7:e7093. doi: 10.7717/peerj.7093 (PMC6612418; doi:10.7717/peerj.7093)
Supplement: Appendix S1 [file peerj-07-7093-s001.docx]

Appendix A. Reservoir and Ponds Information in the SWAT model

| **Sub-basin** | **principle surface area (ha)**  **PND_PSA** | **principle volume (10^4 m3)**  **PND_PVOL** | **emergency spillway surface area (ha)**  **PND_ESA** | **emergency spillway volume (10^4 m3)**  **PND_EVOL** | **drainage area (ha)** | **sub-basin area (ha)** | **drainage area/ sub-basin area**  **PND_FR** |
| --- | --- | --- | --- | --- | --- | --- | --- |
| 1 | 1.52 | 3.05 | 2.29 | 4.57 | 45.7 | 1214.17 | 0.04 |
| 2 | 2.65 | 5.31 | 3.98 | 7.96 | 79.6 | 694.81 | 0.11 |
| 3 | 1.09 | 2.18 | 1.64 | 3.27 | 32.7 | 2112.78 | 0.02 |
| 4 | - | - | - | - | - | - | - |
| 5 | 4.84 | 9.69 | 7.27 | 14.53 | 145.3 | 570.03 | 0.25 |
| 6 | 20 | 40.30 | 30 | 62.45 | 724.5 | 784.94 | 0.92 |
| 7 | 2.06 | 4.11 | 3.08 | 6.17 | 61.7 | 625.04 | 0.10 |
| 8 | 1.93 | 3.86 | 2.90 | 5.79 | 57.9 | 817.80 | 0.07 |
| 9 | 6.29 | 12.59 | 9.44 | 18.88 | 37.74 | 37.74 | 1.00 |
| 10 | 0.84 | 1.67 | 1.26 | 2.51 | 25.1 | 697.89 | 0.04 |
| 11 | 12.62 | 25.24 | 18.93 | 37.86 | 57.45 | 57.45 | 1.00 |
| 12 | 0.04 | 0.07 | 0.06 | 0.11 | 1.1 | 23.70 | 0.05 |
| 13 | 20 | 41.71 | 30 | 62.56 | 925.6 | 2822.14 | 0.33 |
| 14 | 7.54 | 15.09 | 11.31 | 22.63 | 226.3 | 571.50 | 0.40 |
| 15 | 7.94 | 15.89 | 11.92 | 23.83 | 238.3 | 882.17 | 0.27 |
| 16 | 4.37 | 8.73 | 6.55 | 13.10 | 131.0 | 1823.63 | 0.07 |
| 17 | - | - | - | - | - | - | - |
| 18 | 5.37 | 10.73 | 8.05 | 16.10 | 161.0 | 1185.20 | 0.14 |
| 19 | 20 | 40.48 | 30 | 62.73 | 727.3 | 1139.11 | 0.64 |
| 20 | 20 | 40.68 | 30 | 62.02 | 730.2 | 2560.27 | 0.29 |
| 21 |  | - | - | - | - | - | - |
| 22 | 11.80 | 23.60 | 17.70 | 35.40 | 354.0 | 1574.00 | 0.22 |
| 23 | 5.96 | 11.92 | 8.94 | 17.88 | 178.8 | 1596.47 | 0.11 |
| 24 | 1.42 | 2.84 | 2.13 | 4.25 | 42.5 | 773.11 | 0.06 |
| 25 | 8.95 | 17.89 | 13.42 | 26.84 | 268.4 | 1665.47 | 0.16 |
| 26 | 3.77 | 7.53 | 5.65 | 11.30 | 113.0 | 1415.00 | 0.08 |
| 27 | 1.85 | 3.69 | 2.77 | 5.54 | 55.4 | 560.79 | 0.10 |
| 28 | 0.04 | 0.07 | 0.05 | 0.11 | 1.1 | 53.39 | 0.02 |
| 29 | 3.18 | 6.36 | 4.77 | 9.54 | 95.4 | 952.56 | 0.10 |
| 30 | 3.26 | 6.51 | 4.89 | 9.77 | 97.7 | 888.28 | 0.11 |
| 31 | 0.26 | 0.52 | 0.39 | 0.79 | 7.9 | 218.01 | 0.04 |
| 32 | 0.15 | 0.30 | 0.23 | 0.45 | 4.5 | 64.24 | 0.07 |
| 33 | 7.42 | 14.85 | 11.14 | 22.27 | 222.7 | 753.34 | 0.30 |
| 34 | 6.19 | 12.39 | 9.29 | 18.58 | 185.8 | 1328.45 | 0.14 |
| 35 | - | - | - | - | - | - | - |
| 36 | 2.48 | 4.96 | 3.72 | 7.44 | 74.4 | 919.52 | 0.08 |
| 37 | - | - | - | - | - | - | - |
| 38 | - | - | - | - | - | - | - |
| 39 | 1.51 | 3.02 | 2.27 | 4.53 | 45.3 | 904.65 | 0.05 |
| 40 | 0.07 | 0.14 | 0.11 | 0.21 | 2.1 | 217.57 | 0.01 |
| 41 | 1.83 | 3.66 | 2.74 | 5.49 | 54.9 | 655.48 | 0.08 |
| 42 | 0.09 | 0.18 | 0.13 | 0.27 | 2.7 | 198.45 | 0.01 |
| 43 | 2.42 | 4.83 | 3.62 | 7.25 | 72.5 | 748.99 | 0.10 |
