# Supplementary material for: Modeling the impacts of agricultural best management practices on runoff, sediment, and crop yield in an agriculture-pasture intensive watershed
Source: PeerJ. 2019 Jul 4;7:e7093. doi: 10.7717/peerj.7093 (PMC6612418; doi:10.7717/peerj.7093)
Supplement: Appendix S2 [file peerj-07-7093-s002.docx]

Appendix B. Soil characteristics for each soil ID (SSURGO database)

| **MUID** | **SEQN** | **SNAM** | **S5ID** | **Texture** |
| --- | --- | --- | --- | --- |
| 381869 | 508001 | Acme | OK015 | SICL-BR |
| 381870 | 508004 | Grant | OK015 | L-L-SIL-L-BR |
| 381871 | 508011 | Binger | OK015 | FSL-SCL-BR |
| 381872 | 508018 | Binger | OK015 | FSL-SCL-BR |
| 381873 | 508023 | Binger | OK015 | FSL-SCL-BR |
| 381874 | 508024 | Binger | OK015 | FSL-SCL-BR |
| 381875 | 508025 | Binger | OK015 | FSL-SCL-BR |
| 381876 | 508027 | Cyril | OK015 | FSL-L |
| 381879 | 508036 | Darnell | OK015 | FSL-FSL-BR |
| 381881 | 508040 | Dougherty | OK015 | LFS-LFS-SCL-FSL-LFS |
| 381882 | 508045 | Dougherty | OK015 | LFS-LFS-SCL-FSL-LFS |
| 381883 | 508052 | Eufaula | OK015 | FS-FS-FS |
| 381884 | 508058 | Eufaula | OK015 | LFS-FS-FS |
| 381887 | 508073 | Gracemont | OK015 | FSL-FSL-L |
| 381888 | 508077 | Grant | OK015 | L-L-SIL-L-BR |
| 381889 | 508078 | Grant | OK015 | L-L-SIL-L-BR |
| 381890 | 508079 | Grant | OK015 | L-L-SIL-L-BR |
| 381891 | 508080 | Grant | OK015 | L-L-SIL-L-BR |
| 381894 | 508088 | Konawa | OK015 | LFS-SCL-LFS |
| 381895 | 508093 | Konawa | OK015 | LFS-SCL-LFS |
| 381897 | 508095 | Ironmound | OK015 | FSL-L-BR |
| 381898 | 508097 | Ironmound | OK015 | FSL-L-BR |
| 381901 | 508107 | Minco | OK015 | VFSL-SIL-SIL |
| 381902 | 508110 | Minco | OK015 | VFSL-SIL-SIL |
| 381903 | 508111 | Minco | OK015 | SIL-SIL-SIL |
| 381904 | 508112 | Noble | OK015 | FSL-FSL |
| 381905 | 508118 | Noble | OK015 | FSL-FSL |
| 381908 | 508130 | Pond Creek | OK015 | FSL-SICL-L |
| 381909 | 508136 | Pond Creek | OK015 | FSL-SICL-L |
| 381910 | 508142 | Pond Creek | OK015 | SIL-SICL-L |
| 381911 | 508148 | Pond Creek | OK015 | SIL-SICL-L |
| 381912 | 508154 | Pond Creek | OK015 | SIL-SICL-L |
| 381913 | 508155 | Port | OK015 | SIL-SIL-L |
| 381914 | 508162 | Port | OK015 | SIL-SIL-L |
| 381915 | 508168 | Pulaski | OK015 | FSL-FSL-SR LFS L |
| 381916 | 508174 | Ironmound | OK015 | L-L-BR |
| 381918 | 508181 | Minco | OK015 | SIL-SIL-SIL |
| 381920 | 508192 | Darnell | OK015 | FSL-FSL-BR |
| 381921 | 508194 | Lovedale | OK015 | FSL-SCL-SL-S |
| 381922 | 508200 | Lovedale | OK015 | FSL-SCL-SL-S |
| 381928 | 508213 | Water | OK015 | water |
| 381929 | 508214 | Woodward | OK015 | SIL-SIL-BR |
| 382310 | 507114 | Carey | OK039 | SIL-SICL-L-BR |
| 382316 | 507133 | Cornick | OK039 | SIL-BR-BR |
| 382325 | 507160 | Grant | OK039 | L-L-L-L-BR |
| 382326 | 507161 | Hardeman | OK039 | FSL-FSL |
| 382327 | 507162 | Lucien | OK039 | VFSL-VFSL-BR |
| 382328 | 507164 | Minco | OK039 | VFSL-VFSL-VFSL |
| 382332 | 507173 | Pond Creek | OK039 | FSL-SIL-SICL-SIL |
| 382333 | 507179 | Pond Creek | OK039 | SIL-SIL-SICL-SIL |
| 382334 | 507185 | Pond Creek | OK039 | SIL-SIL-SICL-SIL |
| 382339 | 1170380 | Quinlan | OK039 | SIL-SIL-BR |
| 382341 | 507217 | Lovedale | OK039 | FSL-FSL-SCL-FSL |
| 382342 | 507218 | St. Paul | OK039 | SIL-SICL-SICL-SICL-SIL |
| 382343 | 507224 | St. Paul | OK039 | SIL-SICL-SICL-SICL-SIL |
| 382344 | 507225 | St. Paul | OK039 | SIL-SICL-SICL-SICL-SIL |
| 382345 | 507227 | Water | OK039 | water |
| 382348 | 507230 | Woodward | OK039 | SIL-SIL-BR |
| 382349 | 507231 | Woodward | OK039 | SIL-SIL-BR |
| 382350 | 507238 | Woodward | OK039 | SIL-SIL-BR |
| 382351 | 507241 | Quinlan | OK039 | SIL-SIL-BR |
| 384993 | 508521 | Clairemont | OK149 | SIL-SIL |
| 384994 | 508527 | Cordell | OK149 | SICL-SICL-GRV-SICL-BR |
| 384995 | 508528 | Cordell | OK149 | SICL-SICL-GRV-SICL-BR |
| 384996 | 508530 | Cornick | OK149 | SIL-BR-BR |
| 384997 | 508532 | Devol | OK149 | LFS-FSL-LFS |
| 384998 | 508538 | Devol | OK149 | LFS-FSL-LFS |
| 385003 | 508496 | Altus | OK149 | FSL-FSL-SCL-SCL |
| 385004 | 508565 | Dill | OK149 | FSL-FSL-BR |
| 385005 | 508567 | Dill | OK149 | FSL-FSL-BR |
| 385007 | 508575 | Dougherty | OK149 | LFS-LFS-SCL-FSL-LFS |
| 385011 | 508590 | Hardeman | OK149 | FSL-FSL |
| 385012 | 508596 | Hardeman | OK149 | FSL-FSL |
| 385013 | 508597 | Hardeman | OK149 | FSL-FSL |
| 385018 | 508601 | Pond Creek | OK149 | FSL-SICL-SIL |
| 385019 | 508607 | Pond Creek | OK149 | FSL-SICL-SIL |
| 385020 | 508613 | Port | OK149 | SIL-SICL-SIL |
| 385021 | 508619 | Eda | OK149 | LFS-LFS-LFS |
| 385023 | 508622 | Quinlan | OK149 | L-L-BR |
| 385024 | 508624 | Quinlan | OK149 | L-L-BR |
| 385026 | 508626 | Quinlan | OK149 | L-L-BR |
| 385027 | 508628 | Quinlan | OK149 | FSL-L-BR |
| 385028 | 508630 | Reinach | OK149 | SIL-SIL |
| 385030 | 508637 | Lovedale | OK149 | FSL-SCL-FSL-FSL |
| 385031 | 508643 | Lovedale | OK149 | FSL-SCL-FSL-FSL |
| 385032 | 508644 | St. Paul | OK149 | SIL-SICL-SICL-SICL-SIL |
| 385033 | 508650 | St. Paul | OK149 | SIL-SICL-SICL-SIL |
| 385034 | 508656 | St. Paul | OK149 | SIL-SICL-SICL-SIL |
| 385036 | 508511 | Binger | OK149 | FSL-SCL-BR |
| 385037 | 508660 | Woodward | OK149 | SIL-SIL-BR |
| 385038 | 508661 | Woodward | OK149 | SIL-SIL-BR |
| 385039 | 508662 | Woodward | OK149 | SIL-SIL-BR |
| 385040 | 508663 | Woodward | OK149 | SIL-SIL-BR |
| 385041 | 508673 | Woodward | OK149 | L-SIL-BR |
| 385042 | 508675 | Woodward | OK149 | L-SIL-BR |
| 385044 | 508512 | Binger | OK149 | FSL-SCL-BR |
| 385045 | 508513 | Carey | OK149 | SIL-CL-L-BR |
| 385046 | 508514 | Carey | OK149 | SIL-CL-L-BR |
| 385047 | 508515 | Clairemont | OK149 | SIL-SIL |
| 385048 | 508682 | Water | OK149 | water |
