# Supplementary material for: Modeling the impacts of agricultural best management practices on runoff, sediment, and crop yield in an agriculture-pasture intensive watershed
Source: PeerJ. 2019 Jul 4;7:e7093. doi: 10.7717/peerj.7093 (PMC6612418; doi:10.7717/peerj.7093)
Supplement: Appendix S3 [file peerj-07-7093-s003.docx]

Appendix C.1. Conventional (reduced) tillage for dryland crops and pasture

| **Crop** | **Date** | **Operation** |
| --- | --- | --- |
| **Cotton** | 1.1 | Tillage operation (Disk Plow Ge23ft) |
|  | 3.15 | Tillage operation ( Disk Plow Ge23ft) |
|  | 5.15 | Tillage operation (Springtooth Harrow Ge15ft) |
|  | 6.1 | Tillage operation (Finishing Harrow Lt15ft)  Pesticide Operation (Pendimehalin, 0.25 kg) |
|  | 6.10 | Fertilizer application (Elemental Nitrogen, 50 kg) |
|  | 6.11 | Plant |
|  | 7.1 | Tillage operation (Row Cultivator Ge15ft) |
|  | 11.15 | Harvest and kill |
| **Pasture** | 1.1 | Plant |
|  | 3.1 | Auto fertilization |
|  | 5.1 | Grazing operation (Beef-Fresh Manure, GRZ_DAYS^*^: 180, BIO_EAT^*^: 3, BIO_TRMP^*^: 0.47, MANURE_KG^*^: 1.5) |
| **Winter wheat** | 3.15 | Fertilizer application (Elemental Nitrogen, 80 kg) |
|  | 6.1 | Harvest and kill |
|  | 7.1 | Tillage operation (Chisel Plow Gt15ft) |
|  | 8.1 | Tillage operation (Offset Dis/heavduty Ge19ft) |
|  | 9.20 | Fertilizer application (Elemental Nitrogen, 80 kg)  (Elemental Phosphorus, 35 kg) |
|  | 9.22 | Tillage operation (Disk Plow Ge23ft) |
|  | 9.24 | Tillage operation (Springtooth Harrow Lt15ft) |
|  | 9.25 | Plant |
|  | 12.1 | Grazing operation (GRZ_DAYS^*^: 90, BIO_EAT^*^: 3, BIO_TRMP^*^: 0.47, MANURE_KG^*^: 1.5) |
| **Grain sorghum** | 5.1 | Plant |
|  | 5.27 | Fertilizer application (Elemental Nitrogen, 150 kg) |
|  | 5.28 | Tillage operation (Springtooth Harrow Ge15ft, Disk Plow Ge23ft, Mecoprop Amine, 125), Pesticide Operation (Mecoprop Amine, 125 kg) |
|  | 10.18 | Tillage operation (Disk Plow Ge23ft) |
|  | 10.20 | Tillage operation (Springtooth Harrow Ge15ft) |
|  | 10.30 | Harvest and kill |
| **Alfalfa** | 4.1 | Harvest only |
|  | 5.15 | Harvest only |
|  | 7.1 | Harvest only |
|  | 8.29 | Fertilizer application (Elemental Nitrogen, 50 kg), (Elemental Phosphorous, 20) |
|  | 9.7 | Plant |
|  | 10.15 | Harvest only |
| **Hay** | 4.1 | Harvest only |
|  | 7.1 | Harvest only |
|  | 8.29 | Auto fertilization |
|  | 9.7 | Plant |
|  | 10.15 | Harvest only |
| **Rye** | 6.10 | Harvest only |
|  | 8.10 | Fertilizer application (Elemental Nitrogen, 80 kg), (Elemental Phosphorous, 35) |
|  | 9.20 | Plant |
|  | 9.15 | Grazing operation (GRZ_DAYS^*^: 150, BIO_EAT^*^: 3, BIO_TRMP^*^: 0.47, MANURE_KG^*^: 1.5) |

^*^AUTO_NSTRS: Nitrogen stress factor of cover/plant triggers fertilization. This factor ranges from 0.0 to 1.0 where 0.0 indicates there is no growth of the plant due to nitrogen stress and 1.0 indicates there4 is no reduction of plant growth due to nitrogen stress.

^*^GRZ_DAYS: Number of consecutive days grazing takes place in the HRU

^*^BIO_EAT: dry weight of biomass consumed daily ((kg/ha)/day)

^*^ BIO_TRMP: dry weight of biomass trampled daily ((kg/ha)/day)

^*^MANURE_KG: dry weight of manure deposited daily ((kg/ha)/day)

Appendix C.2. Conventional (reduced) tillage for irrigated crops and pasture

| **Crop** | **Date** | **Operation** |
| --- | --- | --- |
| **Cotton** | 1.1 | Tillage operation (Disk Plow Ge23ft) |
|  | 3.15 | Tillage operation ( Disk Plow Ge23ft) |
|  | 5.15 | Tillage operation (Springtooth Harrow Ge15ft) |
|  | 6.1 | Tillage operation (Finishing Harrow Lt15ft)  Pesticide Operation (Pendimehalin, 0.25 kg)  Irrigation operation (IRR_AMT^*^, 33 mm) |
|  | 6.10 | Fertilizer application (Elemental Nitrogen, 50 kg) |
|  | 6.11 | Plant |
|  | 7.1 | Tillage operation (Row Cultivator Ge15ft)  Irrigation operation (IRR_AMT, 33 mm) |
|  | 7.8 till 9.15 (One irrigation per week) | Irrigation operation (IRR_AMT, 33 mm) |
|  | 11.15 | Harvest and kill |
| **Pasture (Bermuda)** | 1.1 | Plant |
|  | 3.1 | Auto fertilization |
|  | 4.1 | Auto irrigation |
|  | 5.1 | Grazing operation (Beef-Fresh Manure, GRZ_DAYS^*^: 180, BIO_EAT^*^: 3, BIO_TRMP^*^: 0.47, MANURE_KG^*^: 1.5) |
| **Winter wheat** | 3.15 | Fertilizer application (Elemental Nitrogen, 80 kg) |
|  | 4.3 | Auto irrigation |
|  | 6.1 | Harvest and kill |
|  | 7.1 | Tillage operation (Offset Dis/heavduty Ge19ft) |
|  | 8.1 | Tillage operation (Chisel Plow Gt15ft) |
|  | 9.20 | Fertilizer application (Elemental Nitrogen, 80 kg)  (Elemental Phosphorus, 35 kg)  Auto irrigation |
|  | 9.22 | Tillage operation (Disk Plow Ge23ft) |
|  | 9.24 | Tillage operation (Springtooth Harrow Lt15ft) |
|  | 9.25 | Plant |
|  | 11.3 | Auto irrigation |
|  | 12.1 | Grazing operation (GRZ_DAYS^*^: 90, BIO_EAT^*^: 3, BIO_TRMP^*^: 0.47, MANURE_KG^*^: 1.5) |
| **Grain sorghum** | 5.1 | Plant |
|  | 5.27 | Fertilizer application (Elemental Nitrogen, 150 kg) |
|  | 5.28 | Tillage operation (Springtooth Harrow Ge15ft, Disk Plow Ge23ft, Mecoprop Amine, 125) |
|  | 6.1 | Auto irrigation initial |
|  | 10.18 | Tillage operation (Disk Plow Ge23ft) |
|  | 10.20 | Tillage operation (Springtooth Harrow Ge15ft) |
|  | 10.30 | Harvest and kill |

*IRR_AMT: Depth of irrigation water applied on HRU (mm)

Appendix C.3. No-till irrigated cotton with winter wheat as cover crop

| **Crop** | **Date** | **Operation** |
| --- | --- | --- |
|  | 03.15 | Fertilizer application (Elemental Nitrogen, 80 kg)  (Elemental Phosphorus, 30 kg) |
|  | 04.03 | Auto Irrigation |
| **Winter wheat** | 06.1 | kill |
|  | 06.2 | Pesticide Operation (Pendimehalin, 0.25 kg) |
|  | 06.03 | Irrigation operation (IRR_AMT, 33 mm) |
|  | 06.10 | Fertilizer application (Elemental Nitrogen, 150 kg) |
| **Cotton** | 06.11 | Plant |
|  | 07.1 till 09.15 one irrigation in per week | Irrigation operation (IRR_AMT, 33 mm) |
| **Cotton** | 11.1 | Harvest and kill |
|  | 11.2 | Fertilizer application (Elemental Nitrogen, 80 kg)  (Elemental Phosphorus, 35 kg) |
|  | 11.2 | Auto Irrigation |
| **Winter wheat** | 11.3 | Plant |
|  | 12.01 | Auto Irrigation |
| **Winter wheat** | 12.20 | Grazing operation (GRZ_DAYS^*^: 90, BIO_EAT^*^: 3, BIO_TRMP^*^: 0.47, MANURE_KG^*^: 1.5) |

Appendix C.4. Rotation of winter wheat with canola in dryland with no-till system

| **Crop** | **Year** | **Date** | **Operation** |
| --- | --- | --- | --- |
| **Winter wheat** | Year 1 | 01.01 | Plant wheat |
|  |  | 06.01 | Harvest and kill |
|  |  | 09.20 | Fertilizer application (Elemental Nitrogen, 80 kg)  (Elemental Phosphorus, 35 kg) |
|  |  | 09.25 | Plant wheat |
|  |  | 12.01 | Grazing operation (GRZ_DAYS^*^: 90, BIO_EAT^*^: 3, BIO_TRMP^*^: 0.47, MANURE_KG^*^: 1.5) |
| **Winter canola** | Year 2 | 03.01 | Fertilizer application (Elemental Nitrogen, 80 kg) |
|  |  | 06.01 | Harvest and kill |
|  |  | 09.20 | Fertilizer application (Elemental Nitrogen, 38 kg)  (Elemental Phosphorus, 15 kg) |
|  |  | 09.25 | Plant winter canola |
| **Winter wheat** | Year 3 | 04.01 | Fertilizer application (Elemental Nitrogen, 76 kg)  (Elemental Phosphorus, 30 kg) |
|  |  | 06.10 | Harvest and kill |
|  |  | 09.01 | Fertilizer application (Elemental Nitrogen, 80 kg)  (Elemental Phosphorus, 35 kg) |
|  |  | 09.25 | Plant wheat |
|  |  | 12.01 | Grazing operation (GRZ_DAYS^*^: 90, BIO_EAT^*^: 3, BIO_TRMP^*^: 0.47, MANURE_KG^*^: 1.5) |
| **Winter canola** | Year 3 | 03.01 | Fertilizer application (Elemental Nitrogen, 80 kg) |
|  |  | 06.01 | Harvest and kill |
|  |  | 09.20 | Fertilizer application (Elemental Nitrogen, 38 kg)  (Elemental Phosphorus, 15 kg) |
|  |  | 09.25 | Plant winter canola |

Appendix C.5. Rotation of winter wheat with irrigated canola with no-till system

| **Crop** | **Year** | **Date** | **Operation** |
| --- | --- | --- | --- |
| **Winter wheat** | Year 1 | 01.01 | Plant wheat |
|  |  | 04.01 | Auto irrigation |
|  |  | 06.01 | Harvest and kill |
|  |  | 08.25 | Auto irrigation |
|  |  | 09.20 | Fertilizer application (Elemental Nitrogen, 80 kg)  (Elemental Phosphorus, 35 kg) |
|  |  | 09.25 | Plant wheat |
|  |  | 11.11 | Auto irrigation |
|  |  | 12.01 | Grazing operation (GRZ_DAYS^*^: 90, BIO_EAT^*^: 3, BIO_TRMP^*^: 0.47, MANURE_KG^*^: 1.5) |
| **Winter canola** | Year 2 | 03.01 | Fertilizer application (Elemental Nitrogen, 80 kg) |
|  |  | 04.03 | Auto irrigation |
|  |  | 06.01 | Harvest and kill |
|  |  | 09.20 | Fertilizer application (Elemental Nitrogen, 38 kg)  (Elemental Phosphorus, 15 kg) |
|  |  | 09.25 | Plant winter canola |
| **Winter wheat** | Year 3 | 04.01 | Fertilizer application (Elemental Nitrogen, 76 kg)  (Elemental Phosphorus, 30 kg) |
|  |  | 06.10 | Harvest and kill |
|  |  | 08.25 | Auto irrigation |
|  |  | 09.01 | Fertilizer application (Elemental Nitrogen, 80 kg)  (Elemental Phosphorus, 35 kg) |
|  |  | 09.25 | Plant wheat |
|  |  | 11.03 | Auto irrigation |
|  |  | 12.01 | Grazing operation (GRZ_DAYS^*^: 90, BIO_EAT^*^: 3, BIO_TRMP^*^: 0.47, MANURE_KG^*^: 1.5) |
| **Winter canola** | Year 3 | 03.01 | Fertilizer application (Elemental Nitrogen, 80 kg) |
|  |  | 04.03 | Auto irrigation |
|  |  | 06.01 | Harvest and kill |
|  |  | 09.20 | Fertilizer application (Elemental Nitrogen, 38 kg)  (Elemental Phosphorus, 15 kg) |
|  |  | 09.25 | Plant winter canola |

Appendix C.6. Cover cropping of winter wheat with grain sorghum in dryland with no-till system

| **Crop** | **Year** | **Date** | **Operation** |
| --- | --- | --- | --- |
| **Winter wheat** | Year 1 | 01.01 | Plant wheat |
|  |  | 06.01 | Harvest and kill |
|  |  | 10.01 | Fertilizer application (Elemental Nitrogen, 80 kg)  (Elemental Phosphorus, 35 kg) |
|  |  | 10.01 | Plant wheat |
|  |  | 12.01 | Grazing operation (GRZ_DAYS^*^: 90, BIO_EAT^*^: 3, BIO_TRMP^*^: 0.47, MANURE_KG^*^: 1.5) |
|  | Year 2 | 03.15 | Fertilizer application (Elemental Nitrogen, 80 kg) |
|  |  | 06.01 | Harvest and kill |
| **Grain Sorghum, Winter wheat** | Year 3 | 05.01 | Fertilizer application (Elemental Nitrogen, 150 kg) |
|  |  | 05.01 | Plant grain sorghum |
|  |  | 09.30 | Harvest and kill |
|  |  | 10.01 | Fertilizer application (Elemental Nitrogen, 80 kg)  (Elemental Phosphorus, 35 kg)  Plant wheat |
|  |  | 12.01 | Grazing operation (GRZ_DAYS^*^: 90, BIO_EAT^*^: 3, BIO_TRMP^*^: 0.47, MANURE_KG^*^: 1.5) |
|  | Year 4 | 03.15 | Fertilizer application (Elemental Nitrogen, 80 kg) |
|  |  | 06.01 | Harvest and kill |

AppendixC.7. Cover cropping of winter wheat with irrigated grain sorghum with no-till system

| **Crop** | **Year** | **Date** | **Operation** |
| --- | --- | --- | --- |
| **Winter wheat** | Year 1 | 01.01 | Plant wheat |
|  |  | 06.01 | Harvest and kill |
|  |  | 10.01 | Fertilizer application (Elemental Nitrogen, 80 kg)  (Elemental Phosphorus, 35 kg) |
|  |  | 10.01 | Plant wheat |
|  |  | 12.01 | Grazing operation (GRZ_DAYS^*^: 90, BIO_EAT^*^: 3, BIO_TRMP^*^: 0.47, MANURE_KG^*^: 1.5) |
|  | Year 2 | 03.15 | Fertilizer application (Elemental Nitrogen, 80 kg) |
|  |  | 04.01 | Auto irrigation |
|  |  | 06.01 | Harvest and kill |
| **Grain Sorghum, Winter wheat** | Year 3 | 05.01 | Fertilizer application (Elemental Nitrogen, 150 kg) |
|  |  | 05.01 | Plant grain sorghum |
|  |  | 06.01 | Auto irrigation |
|  |  | 09.30 | Harvest and kill |
|  |  | 10.01 | Fertilizer application (Elemental Nitrogen, 80 kg)  (Elemental Phosphorus, 35 kg)  Plant wheat |
|  |  | 12.01 | Grazing operation (GRZ_DAYS^*^: 90, BIO_EAT^*^: 3, BIO_TRMP^*^: 0.47, MANURE_KG^*^: 1.5) |
|  | Year 4 | 03.15 | Fertilizer application (Elemental Nitrogen, 80 kg) |
|  |  | 04.01 | Auto irrigation |
|  |  | 06.01 | Harvest and kill |

Appendix C.8. Cover cropping of winter wheat with cotton in dryland with no-till system

| **Crop** | **Year** | **Date** | **Operation** |
| --- | --- | --- | --- |
| **Winter wheat** | 1 | 09.20 | Fertilizer application (Elemental Nitrogen, 80 kg)  (Elemental Phosphorus, 35 kg) |
|  |  | 09.25 | Plant |
|  |  | 12.01 | Grazing operation (GRZ_DAYS^*^: 90, BIO_EAT^*^: 3, BIO_TRMP^*^: 0.47, MANURE_KG^*^: 1.5) |
| **Cotton** | 2 | 03.15 | Fertilizer application (Elemental Nitrogen, 80 kg) |
|  |  | 06.01 | Harvest and kill wheat |
|  |  | 06.02 | Pesticide Operation (Pendimehalin, 0.25 kg) |
|  |  | 06.10 | Fertilizer application (Elemental Nitrogen, 50 kg) |
|  |  | 06.11 | Plant |
|  |  | 11.04 | Harvest and kill cotton |

Appendix C.9. Cover cropping of winter wheat with irrigated cotton with no-till system

| **Crop** | **Year** | **Date** | **Operation** |
| --- | --- | --- | --- |
| **Winter wheat** | 1 | 09.20 | Fertilizer application (Elemental Nitrogen, 80 kg)  (Elemental Phosphorus, 35 kg)  Auto Irrigation |
|  |  | 09.25 | Plant |
|  |  | 11.03 | Auto Irrigation |
|  |  | 12.01 | Grazing operation (GRZ_DAYS^*^: 90, BIO_EAT^*^: 3, BIO_TRMP^*^: 0.47, MANURE_KG^*^: 1.5) |
| **Cotton** | 2 | 03.15 | Fertilizer application (Elemental Nitrogen, 80 kg) |
|  |  | 04.3 | Auto Irrigation |
|  |  | 06.01 | Harvest and kill wheat |
|  |  | 06.02 | Pesticide Operation (Pendimehalin, 0.25 kg)  Irrigation operation (IRR_AMT, 33 mm) |
|  |  | 06.10 | Fertilizer application (Elemental Nitrogen, 50 kg) |
|  |  | 06.11 | Plant |
|  |  | 07.1 till 09.15 one irrigation in per week | Irrigation operation (IRR_AMT, 33 mm) |
|  |  | 11.04 | Harvest and kill cotton |
