# Supplementary material for: Modeling the impacts of agricultural best management practices on runoff, sediment, and crop yield in an agriculture-pasture intensive watershed
Source: PeerJ. 2019 Jul 4;7:e7093. doi: 10.7717/peerj.7093 (PMC6612418; doi:10.7717/peerj.7093)
Supplement: Appendix S4 [file peerj-07-7093-s004.docx]

Appendix D. Global sensitivity analysis results of SWAT-CUP for streamflow

| **Parameter** | **Description** | **t-stat** | **p-value** |
| --- | --- | --- | --- |
| 6:R__CN2.mgt | SCS Curve number adjustment for soil moisture condition II | 72.99 | 0.00 |
| 8:V__ESCO.hru | Soil evaporation compensation factor | -55.38 | 0.00 |
| 5:V__GW_DELAY.gw | Groundwater delay [Days] | 51.67 | 0.00 |
| 4:V__RCHRG_DP.gw | Deep aquifer percolation fraction | 17.20 | 0.00 |
| 16:V__CH_N2.rte | Manning’s n value for the main channel | -12.20 | 0.00 |
| 15:R__SOL_AWC(..).sol | Available water capacity of soil layer (mm H2O/mm soil) | 9.43 | 0.00 |
| 14:V__ALPHA_BNK.rte | base flow alpha factor for bank | 3.32 | 0.00 |
| 10:V__CH_K1.sub | Effective hydraulic conductivity in tributary channel alluvium ((mmhr-1)) | -2.88 | 0.00 |
| 13:V__TRNSRCH.bsn | Fraction of transmission losses partitioned to deep aquifer | 2.51 | 0.01 |
| 7:V__ALPHA_BF.gw | Baseflow Alpha Factor [Days] | 2.09 | 0.04 |
| 3:V__REVAPMN.gw | Threshold depth of water in the shallow aquifer for "revap" to occur [mm] | -1.06 | 0.29 |
| 12:V__EVRCH.bsn | reach evaporation adjustment factor | -0.96 | 0.34 |
| 17:V__CH_K2.rte | Main channel conductivity | -0.83 | 0.41 |
| 9:V__EPCO.bsn | Plant uptake compensation factor | 0.75 | 0.46 |
| 11:V__SURLAG.bsn | Surface runoff lag coefficient | -0.63 | 0.53 |
| 1:V__GWQMN.gw | Threshold depth of water in the shallow aquifer required for return flow to occur (mm) | 0.60 | 0.55 |
| 2:V__GW_REVAP.gw | Groundwater "revap" coefficient | 0.14 | 0.89 |
