# Supplementary material for: Modeling the impacts of agricultural best management practices on runoff, sediment, and crop yield in an agriculture-pasture intensive watershed
Source: PeerJ. 2019 Jul 4;7:e7093. doi: 10.7717/peerj.7093 (PMC6612418; doi:10.7717/peerj.7093)
Supplement: Appendix S5 [file peerj-07-7093-s005.docx]

Appendix E.1. Cotton yield calibration parameters

| **Parameter** | **Parameter definition** | **Default value** | **Calibrated value** |
| --- | --- | --- | --- |
| BIO_E [(kg/ha)/(MJ/m^2^)] | Radiation use efficiency or biomass energy ratio | 15 | 14 |
| USLE_C | Minimum value of USLE C factor for water erosion | 0.2 | 0.1 |
| HVSTI [(kg/ha)/( kg/ha)] | Harvest index for optimal growing season | 0.4 | 0.3 |
| OV_N | Manning’s “n” value for overland flow | 0.14 | 0.12 |
| BLAI (m^2^/m^2^) | Maximum potential leaf area index | 4 | 3 |
| FRGRW1(fraction) | Fraction of plant growing season to the first point on the optimal leaf area development curve | 0.15 | 0.14 |
| FRGRW2 (fraction) | Fraction of plant growing season to the second point on the optimal leaf area development curve | 0.5 | 0.3 |
| LAIMX1 (fraction) | Fraction maximum leaf area index to the first point on the optimal leaf area development curve | 0.01 | 0.005 |
| CNYLD (kg N/kg seed) | Normal fraction of nitrogen in yield | 0.015 | 0.018 |
| CPYLD (kg P/kg seed) | Normal fraction of Phosphorus in yield | 0.0025 | 0.0027 |

Appendix E.2. Wheat, pasture, and grain sorghum yield calibration parameters

| **Parameter** | **Winter wheat** | | **Pasture** | | **Grain sorghum** | |
| --- | --- | --- | --- | --- | --- | --- |
|  | **Default value** | **Calibrated value** | **Default value** | **Calibrated value** | **Default value** | **Calibrated value** |
| BIO_E [(kg/ha)/(MJ/m^2^)] | 30 | 29 | 35 | 28 | 33.5 | 37 |
| USLE_C | 0.03 | 0.02 | 0.003 | 0.003 | 0.2 | 0.2 |
| HVSTI [(kg/ha)/( kg/ha)] | 0.4 | 0.3 | 0.8 | 0.8 | 0.45 | 0.3 |
| OV_N | 0.14 | 0.12 | 0.3 | 0.25 | 0.14 | 0.12 |
| BLAI (m^2^/m^2^) | 4 | 3 | 4 | 2.5 | 3 | 4.5 |
| FRGRW1(fraction) | 0.05 | 0.03 | 0.05 | 0.03 | 0.15 | 0.15 |
| FRGRW2 (fraction) | 0.45 | 0.35 | 0.49 | 0.35 | 0.5 | 0.5 |
| LAIMX1 (fraction) | 0.05 | 0.03 | 0.05 | 0.03 | 0.05 | 0.05 |
| CNYLD (kg N/kg seed) | 0.025 | 0.02 | 0.0234 | 0.0134 | 0.0199 | 0.02 |
| CPYLD (kg P/kg seed) | 0.0022 | 0.0018 | 0.0033 | 0.0022 | 0.0044 | 0.0032 |
