# Supplementary material for: Modeling the impacts of agricultural best management practices on runoff, sediment, and crop yield in an agriculture-pasture intensive watershed
Source: PeerJ. 2019 Jul 4;7:e7093. doi: 10.7717/peerj.7093 (PMC6612418; doi:10.7717/peerj.7093)
Supplement: Appendix S6 [file peerj-07-7093-s006.docx]

Appendix F. Definition of modeling of crop rotation in SWAT

In rotation calculation, half of each HRU area was considered as one of the rotation crops and it was assumed that each year both crops would be planted in half of the field and the average of the sediment and surface runoff for those two scenarios was used in that year.
